# Nutritional and reproductive signaling revealed by comparative gene expression analysis in *Chrysopa pallens* (Rambur) at different nutritional statuses

**DOI:** 10.1371/journal.pone.0180373

**Published:** 2017-07-06

**Authors:** Benfeng Han, Shen Zhang, Fanrong Zeng, Jianjun Mao

**Affiliations:** Key Laboratory for Biology of Plant Diseases and Insect Pests, Ministry of Agriculture, Institute of Plant Protection, Chinese Academy of Agricultural Sciences, Beijing, China; Inha University, REPUBLIC OF KOREA

## Abstract

**Background:**

The green lacewing, *Chrysopa pallens* Rambur, is one of the most important natural predators because of its extensive spectrum of prey and wide distribution. However, what we know about the nutritional and reproductive physiology of this species is very scarce.

**Results:**

By cDNA amplification and Illumina short-read sequencing, we analyzed transcriptomes of *C*. *pallens* female adult under starved and fed conditions. In total, 71236 unigenes were obtained with an average length of 833 bp. Four vitellogenins, three insulin-like peptides and two insulin receptors were annotated. Comparison of gene expression profiles suggested that totally 1501 genes were differentially expressed between the two nutritional statuses. KEGG orthology classification showed that these differentially expression genes (DEGs) were mapped to 241 pathways. In turn, the top 4 are ribosome, protein processing in endoplasmic reticulum, biosynthesis of amino acids and carbon metabolism, indicating a distinct difference in nutritional and reproductive signaling between the two feeding conditions.

**Conclusions:**

Our study yielded large-scale molecular information relevant to *C*. *pallens* nutritional and reproductive signaling, which will contribute to mass rearing and commercial use of this predaceous insect species.

## Introduction

In recent years, there has been increasing interest in mass production and commercial use of predatory and parasitic natural enemies in biological control of arthropod pests. The green lacewing *Crysopa pallens* is one of the most important natural predators of aphids, coccids, thrips, mites and caterpillars [1–3]. However, very little is known about the nutritional and reproductive signaling in this natural enemy insect.

Vitellogenin (Vg) is the main source of nutrition for the embryo development and is crucial for reproduction of oviparous animals, such as insects [4]. Several hormones, like juvenile hormone (JH), ecdysone and neuropeptides, are involved in regulation of *Vg* gene expression [5–8]. All these endocrine hormones function through certain conserved signaling pathways. There are mainly two pathways involved in conveying of insect nutritional signals, the target of rapamycin (ToR) pathway and the insulin-like peptides (ILPs) pathway [5,9].

ILPs in insects are analogously to both insulin and IGF in vertebrates. So far, genes encoding ILPs have been cloned in species from different insect orders, including Orthoptera, Diptera, Lepidoptera and Hymenoptera [5,10]. Genetic studies revealed that insect ILPs act through a conserved insulin signaling pathway and regulate development, longevity, metabolism, diapause and reproduction [11–19].

The ILP/ToR pathways sense nutrient level and play crucial roles in determining the tradeoff between survival and reproduction [7]. To date, the main elements involved in the ILP/ToR pathways have been identified. When insect reached a nutritional status suitable for inducing reproductive processes, the ILPs are secreted in response to the high nutritional level. Binding of ILPs with the insulin receptor (InR) activates expression of phosphatidylinositol 3-kinase (PI3K) and subsequently increases the production of phosphatidylinositol trisphosphate (PIP3). Acting as a second messenger, the PIP3 recruits Akt (serine/threonine-protein kinase) to plasma membrane. After phosphorylation, the Akt in turn phosphorylates a series of downstream targets to realize pathway function [20,21].

FoxO, the transcription factor forkhead-box, class O, is a pivotal agent relevant to the transcriptional activities of the ILPs pathway [22–24]. In fed condition, FoxO was phosphorylated and exported from nucleus to cytoplasm. In contrast, under starved condition, unphosphorylated FoxO is restricted to the nucleus. Both ILP- and ToR-dependent signals coordinately control the translational effector S6 kinase (S6K) and translation initiation factor 4E-binding protein (4E-BP). In presence of ample nutrients, ILP and ToR signaling become active and lead to phosphorylation of S6K, which promote protein synthesis and growth. In the absence of nutrients, ILP/ToR signaling is attenuated. 4E-BP is hypophosphorylated and associates tightly with eIF4E, leading to reduction of protein synthesis and inhibition of growth [21,23,25,26].

In recent years, deep sequencing via next-generation high throughput techniques has been widely used to gain extensive information about genomes and gene expression profiling. For instance, a normalized transcriptome of *C*. *pallens* was sequenced to facilitate identifying sets of genes involved in olfaction [27]. Here, we constructed two whole-body cDNA libraries in starved and fed conditions and performed Illumina sequencing to gain insight into the expression profiling involved in nutritional and hormone regulation of *C*. *pallens* female reproduction. As a whole, 71236 distinct unigenes were identified and 1501 unigenes were differently expressed between the two nutritional stations. *C*. *pallens Vgs*, digestive enzyme encoding genes, main components of ILP/ToR pathways such as *ILPs*, *InRs*, etc. showed different expression profiles with their counterparts in other insect species. These results provide a good molecular base for further exploring of nutritional metabolism and reproductive regulation in *C*. *pallens* and will contribute to mass rearing of this predatory species.

## Results

### Illumina sequencing and read assembly

The Illumina sequencing of starved and fed female adults produced 93626805 and 88303436 raw reads, respectively. After cleaning and quality check, 92982067 and 87730137 (SRA accession number SRR4181653 and SRP107706) clean reads composed of 27985415270 and 26330193355 nucleotides were obtained, respectively. The sequences from the two libraries were combined and a total of 71236 unigenes were finally obtained (Table 1).

**Table 1:** Summary of *C*. *pallens* transcripomes.

Parameters	Starved	Fed
Total number of raw reads	93626805	88303436
Total number of clean reads	92982067	87730137
Total nucleotides	27985415270	26330193355
Total clear nucleotides	27792700580	26159247872
Q20 percentage (%)	95.7%	95.3%
Q30 percentage (%)	90%	29%
Total number of unigenes	71236	
Mean length of unigenes (nt)	833	
Range in length of unigenes (nt)	202~27462	
N50 of unigenes (nt)	1615	
GC percentage (%)	34.68%	
Total number of genes	71236	
N50 of genes (nt)	1615	
GC percentage (%)	34.68%	

### Gene identification and function annotation

**Fig 1:**
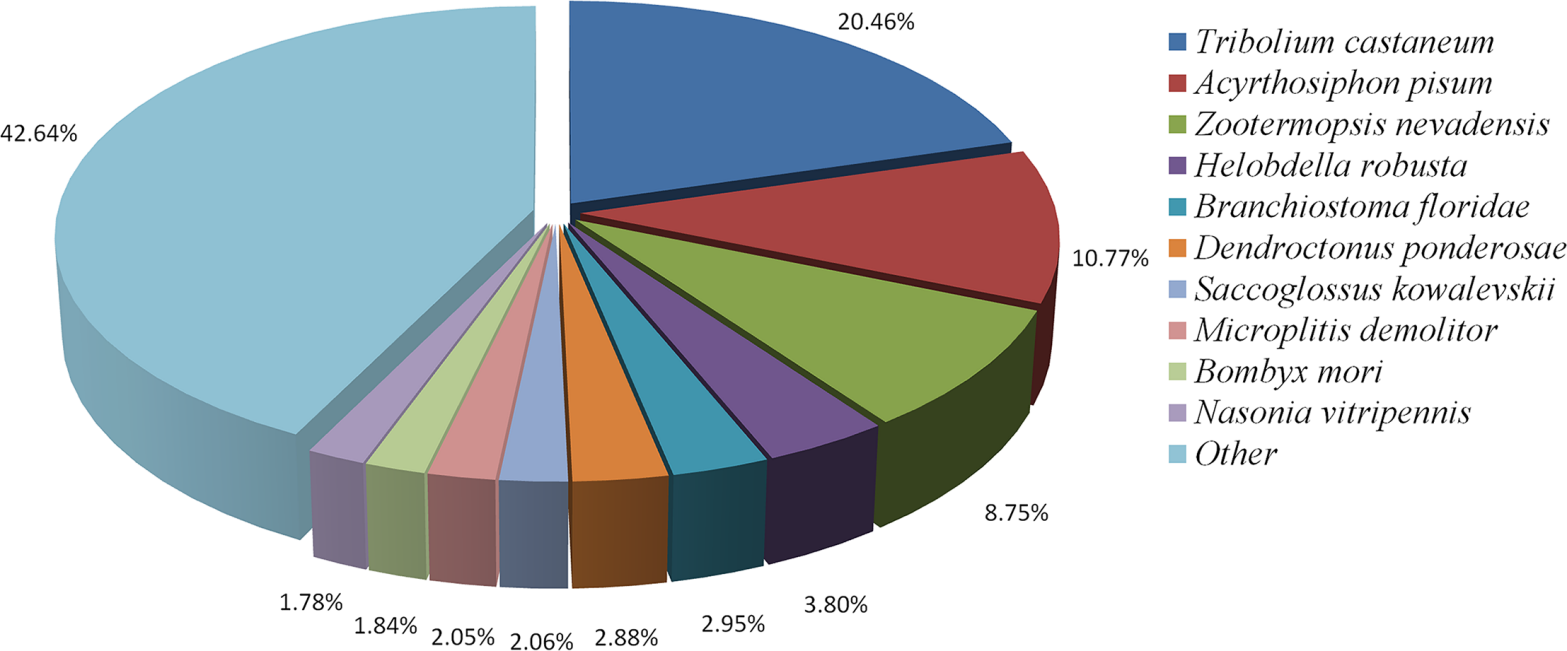
Species distribution of unigene BLASTX results. Unigenes were searched against the NR protein database using BLASTX tool with a cutoff e-value <10^−5^. Proportions of each species were represented by different colors and graphed. Species with proportions less than 1% were not shown.

For functional annotation, the 71236 unigenes were analyzed using BLASTx against NR (Non-Redundant), SwissProt, KEGG (Kyoto Encyclopedia of Genes and Genomes), COG (Clusters of Orthologous Groups) and GO (Gene Ontology) databases with a cut-off e-value of 10^−5^. Totally, 22272 (31.27%) unigenes yield significant BLAST hits. Among them, 19184 (26.93%) were annotated by NR database. The species distribution of the best match for each sequence was shown in Fig 1. The *C*. *pallens* sequences revealed maximum matches of 20.46% with *Tribolium castaneum*, followed by 10.77% with *Acyrthosiphon pisum*.

**Fig 2:**
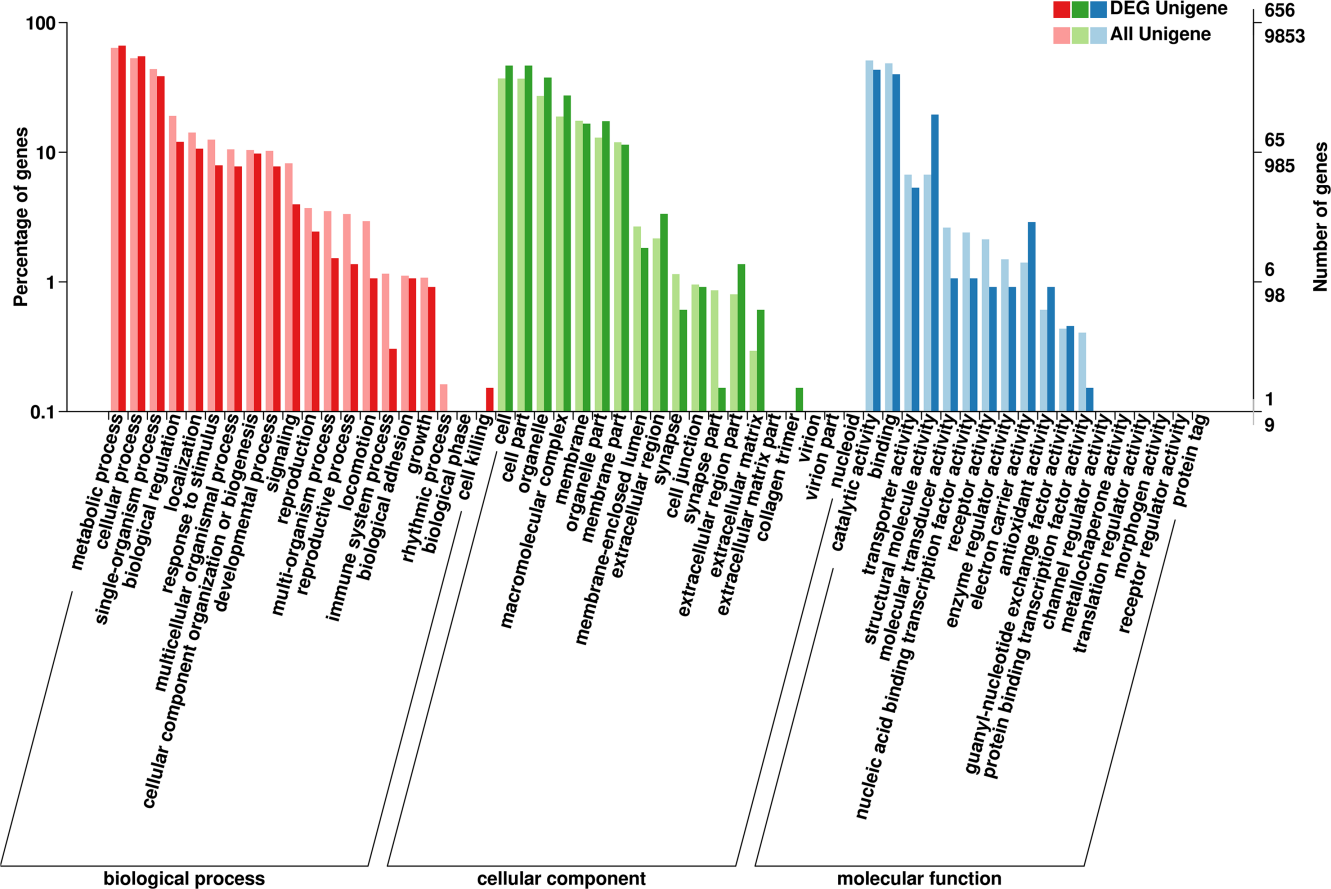
Distribution of differentially expressed genes annotated by GO subclasses. The X-axis shows three GO categories and subcategories. The Y-axis indicates numbers of differentially expressed genes.

The function of the *C*. *pallens* unigenes was classified by GO annotation. Among the annotated unigenes, 9853 (13.83%) corresponded to at least one GO term. A total of 6776, 4706 and 8466 unigenes were involved in the categories of biological process, cellular component and molecular function, respectively. Within the biological process, the three most common categories were metabolic process, cellular process and single-organism process. The three most abundant categories in cellular component were cell, cell part and organelle. In the molecular function, catalytic activity represented the most common GO term (Fig 2).

To investigate the biological pathways of the unigenes, all of the sequences were assigned to the reference canonical pathways in the KEGG. Unigenes in the adult transcriptomes of *C*. *pallens* were mapped to a total of 241 KEGG pathways and the top 20 of them were shown in Table 2.

**Table 2:** The top 20 KEGG pathways in the *C*. *pallens* transcriptomes.

KEGG Pahtway	DEGs_in_Pathway	P-value
Ribosome	113	0.00E+00
Protein processing in endoplasmic reticulum	39	1.47E-02
Biosynthesis of amino acids	30	1.63E-03
Carbon metabolism	28	2.18E-01
Oxidative phosphorylation	28	6.05E-01
Glycolysis / Gluconeogenesis	22	1.31E-02
Phagosome	21	4.75E-01
Lysosome	19	3.54E-01
Glutathione metabolism	17	4.58E-03
RNA transport	16	9.37E-01
Tuberculosis	14	5.18E-02
Drug metabolism—cytochrome P450	13	2.72E-02
Metabolism of xenobiotics by cytochrome P450	13	3.47E-02
Purine metabolism	13	9.02E-01
RNA degradation	11	2.08E-01
FoxO signaling pathway	11	3.70E-01
Antigen processing and presentation	10	1.16E-03
Leishmaniasis	10	2.35E-02
Herpes simplex infection	10	3.87E-02
ECM-receptor interaction	10	5.95E-02

### Sequence alignment and phylogenetic analysis

**Fig 3:**
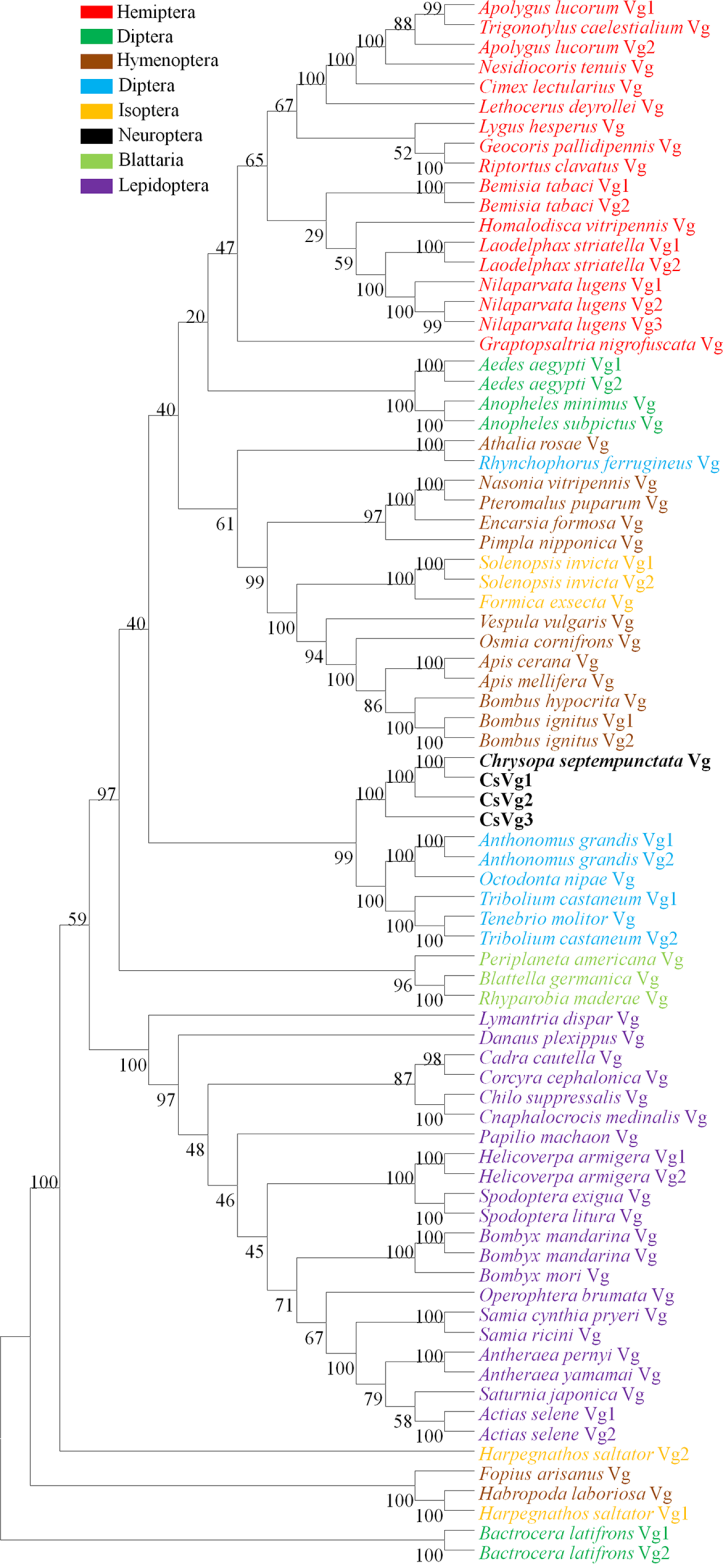
Phylogenetic tree of vitellogenins (Vgs). Vg amino acid sequences from *C*. *pallens* and 62 other insect species were used for phylogenetic analysis. Numbers at thenodes are bootstrap values as percentage. Only bootstrap values above 70 are shown.

**Fig 4:**
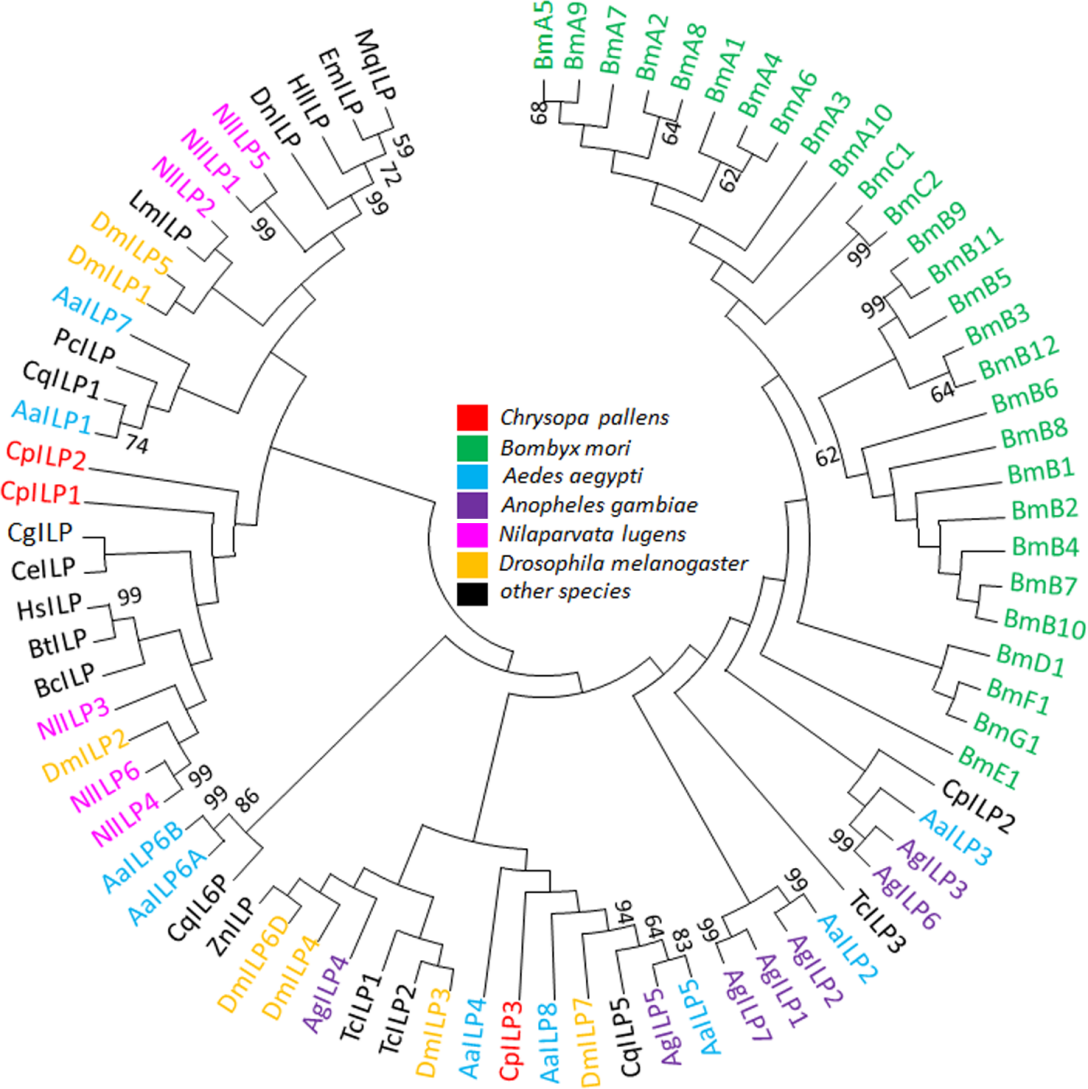
Phylogenetic tree of insulin-like peptides (ILPs) and insulin. Ha: *Homo sapiens*, Aa: *Aedes aegypti*, Ag: *Anopheles gambiae*, Bc: *Branchiostoma californiense*, Bm: *Bombyx mori*, Bt: *Bos taurus*, Ce: *Caenorhabditis elegans*, Cg: *Crassostrea gigas*, Cp: *Culex pipiens*, Cq:*Culex quinquefasciatus*, Dm: *Drosophila melanogaster*, Dn: *Dufourea novaeangliae*, Em: *Eufriesea Mexicana*, Hl: *Habropoda laboriosa*, Hs: *Homo sapiens*, Lm: *Locusta migratoria*, Mq: *Melipona quadrifasciata*, Nl: *Nilaparvata lugens*, Pc: *Priapulus caudatus*, Zn: *Zootermopsis nevadensis*.

The *C*. *pallens* transcriptomes were screened for *Vg* and main components in ILP/ToR signaling pathway. Totally, five unigenes were identified as Vg-encoding sequences. Among them, CK1.comp19582_c0_seq1 showed a 99.75% similarity with the 3040–5433 bp of the first *Vg* (GenBank accession number: JX286617.1) characterized in *C*. *pallens* and was named *C*. *pallens Vg1*. CL242Contig1 is a complete opening reading frame and shows an identity of 97.51% with JX286617.1 and was named *C*. *pallens Vg2*. The other three unigenes, which also exhibit high identity with JX286617.1, are partial mRNA sequences. Because there are no overlaps between every two of them, primers were designed to amplify the gap. PCR results confirmed that they belong to the same *Vg*, *C*. *pallens Vg3*. So, 3 different *Vg* genes were finally identified from the *C*. *pallens* transcriptomes, in total. The 3 *C*. *pallens* Vgs and 75 Vgs from other species were phylogenetically analyzed. The 3 *C*. *pallens* Vgs clustered toghther and were most closely related to Coleoptera Vgs (Fig 3). 3 distinct unigenes encoding ILPs were identified by BLASTX. All of them exhibited typical ILP characteristics, including 4 Cysteines in A chain and 2 Cysteines in B chain. ILP1 and ILP2 contain signal peptides as predicted by SignalP 4.1, but ILP3 contains none. The 3 ILPs from *C*. *pallens* and 76 Vgs from other species were used to construct a phylogenetic tree, in which most ILPs of the same species formed monophyletic groups. The 3 *C*. *pallens* ILPs did not cluster together with a bootstrap percentage>70 (Fig 4).

**Fig 5:**
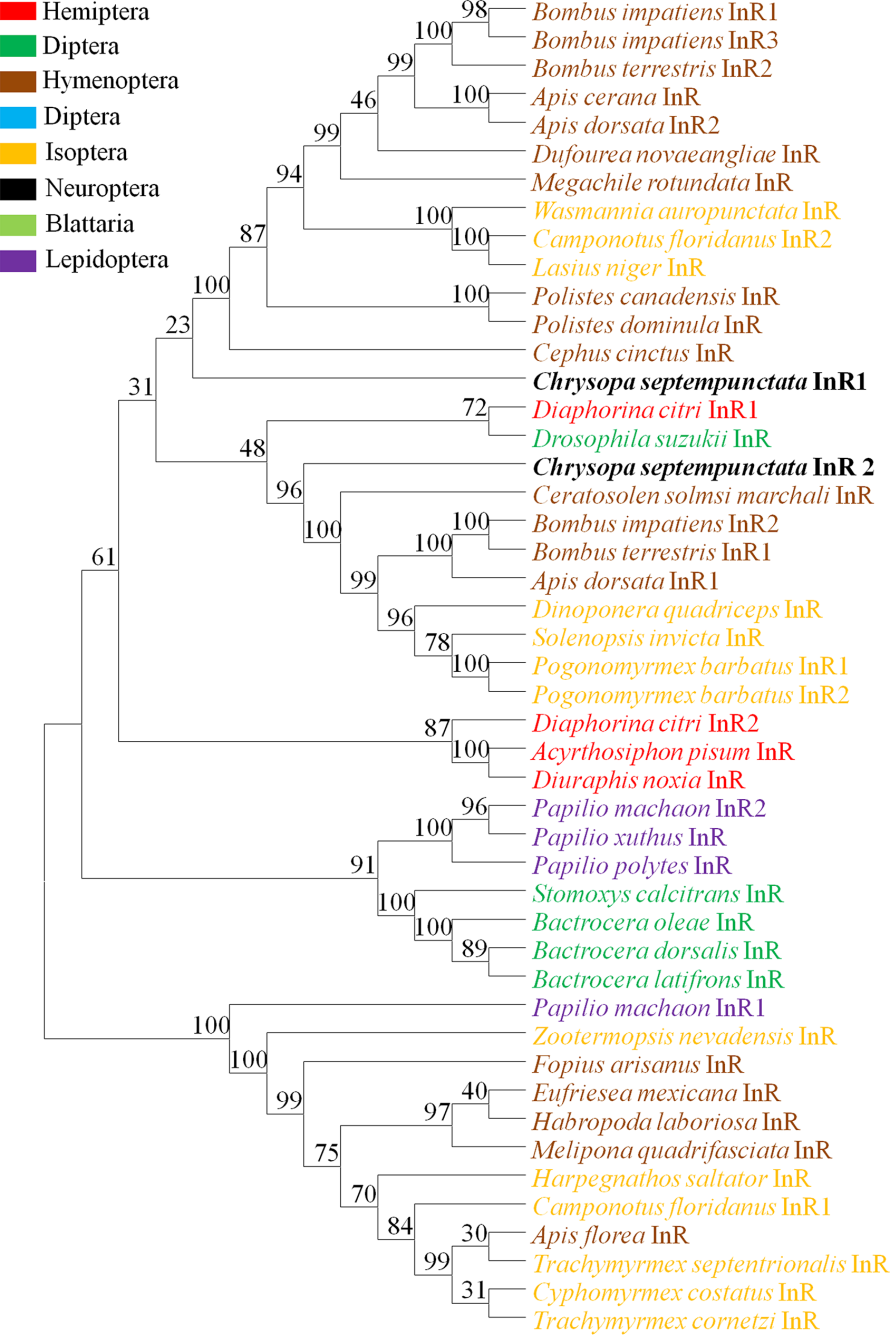
Phylogenetic tree of insulin receptors (InRs). InR amino acid sequences from 47 insect species were used for phylogenetic analysis. Numbers at the nodes are bootstrap values as percentage. Only bootstrap values above 70 are shown.

Two unigenes from the *C*. *pallens* transcriptomes were identified as *InR*. The 2 *C*. *pallens* InRs were phylogenetically analyzed together with 47 InRs from other species. In the phylogenetic tree, *C*. *pallens* InR1 clustered together with *Bactrocera latifrons* InR, but *C*. *pallens* InR2 showed higher homology to *Diaphorina citri* InR1 and *Diuraphis noxia* InR (Fig 5).

In addition, *Akt*, *PI3K*, *Rheb* (*Ras homolog enriched in brain*), *Pten* (*Phosphatase and tensin homology deleted on chromosome ten*), *ToR*, *TSC1*(*Tuberous Sclerosis Complex 1*), *TSC2* (*Tuberous Sclerosis Complex 2*), *S6K*, *4E-BP*, etc. were also identified from the *C*. *pallens* transcriptomes.

### Differences in gene expression between two nutritional situations

**Fig 6:**
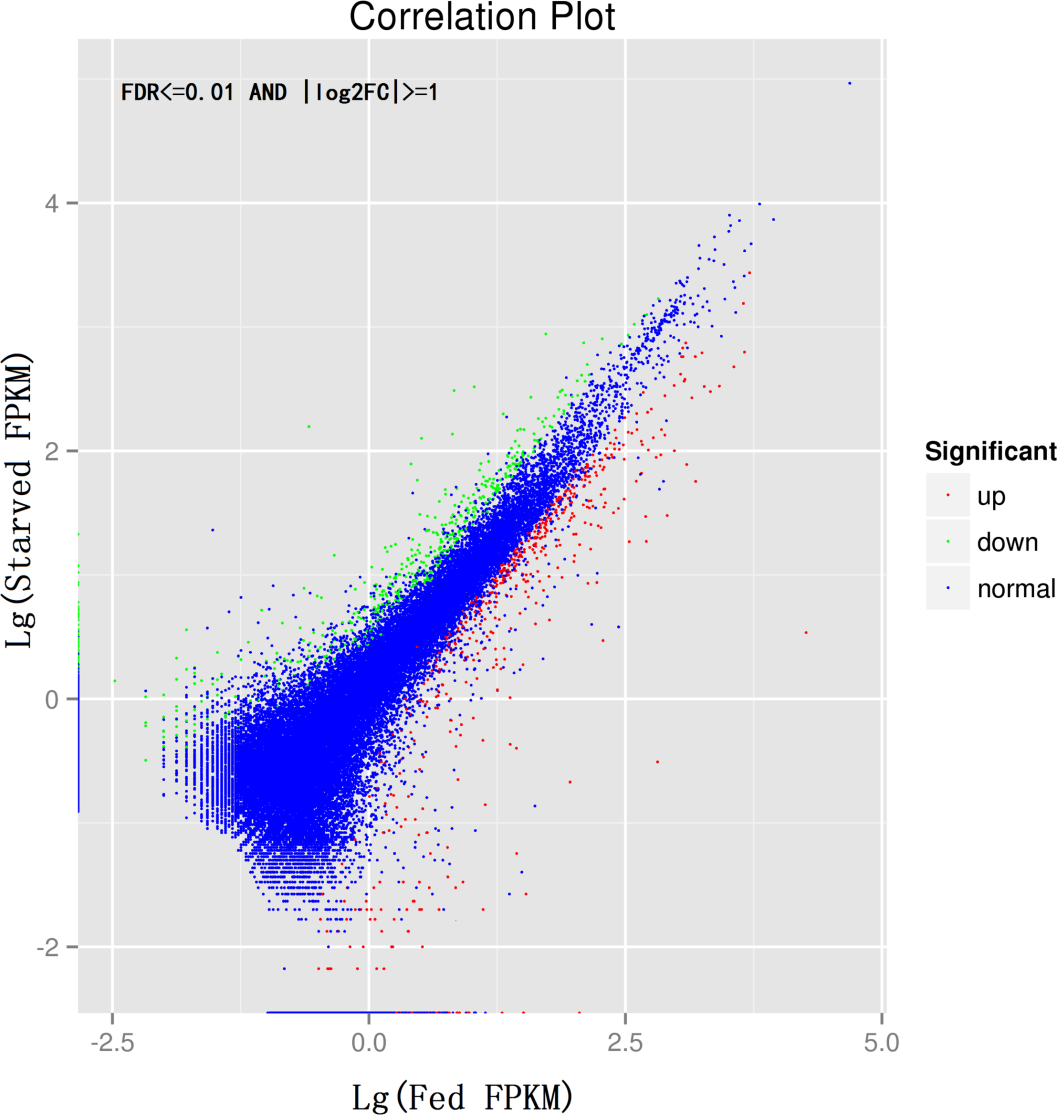
Bitmap of differentially expressed genes. Red and green points mean up-regulated and down-regulated genes in fed female adult, respectively. Blue points represent genes with no expression difference based on criteria of the false discovery rate (FDR) ≤0.001 and an absolute value of the log_2_ ratio≥1.

**Fig 7:**
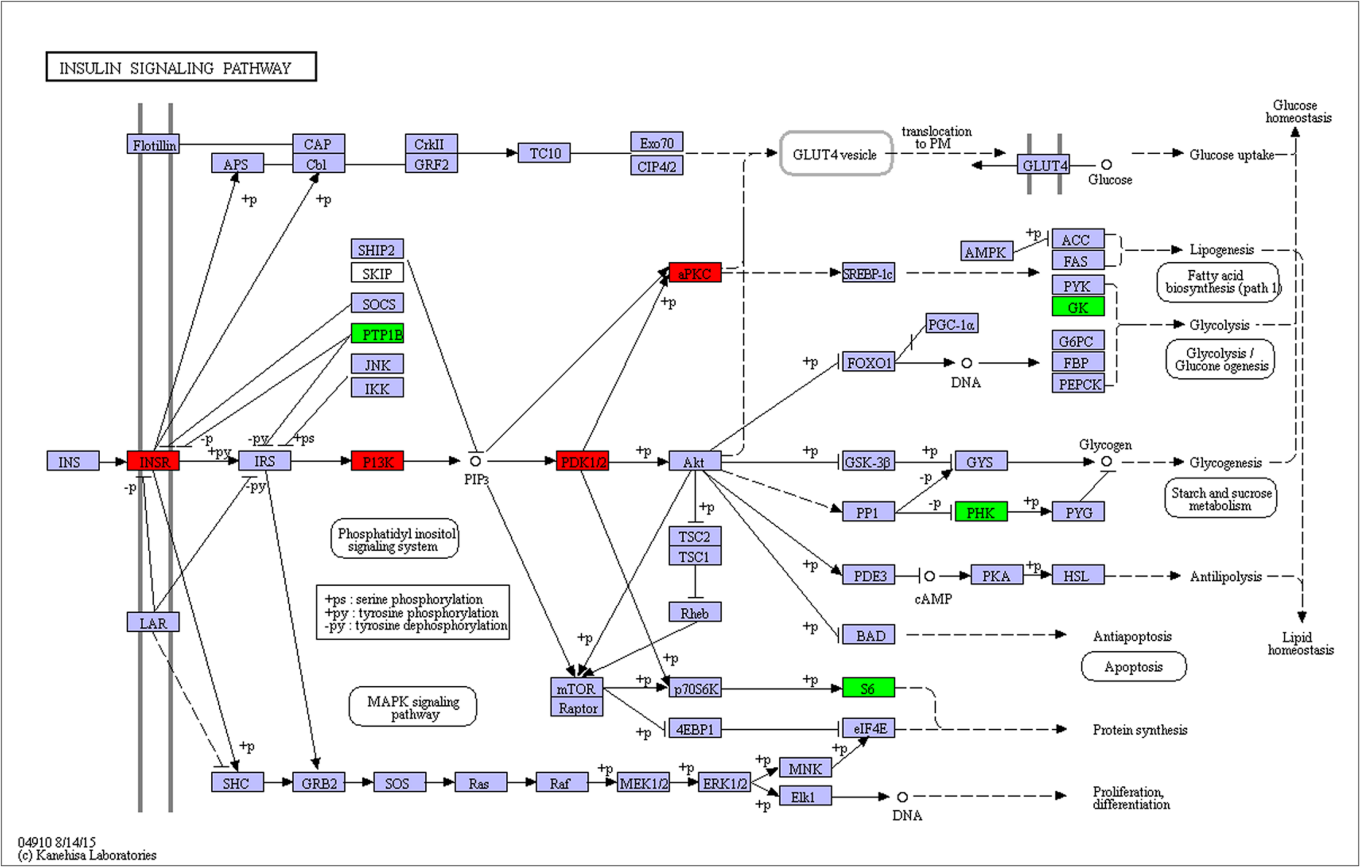
Insulin signaling pathway affected by nutritional status. Red and green backgrounds indicate genes that were up-, down-regulated, respectively, in fed female compared to starved female.

To screen differentially expressed genes in *C*. *pallens* in starved and fed conditions, the number of fregmants mapped to each gene was calculated and normalized by fragments per kb per million fragments (RPKM). Results revealed 1501 genes with significantly different expression abundance. Of these, 837 were up-regulated in fed adult female (Fig 6). The detected fold changes (log_2_ ratio) ranged from minus infinity to plus infinity. These differentially expressed genes (DEGs) were mapped to a total of 241 KEGG pathways and the top 20 of them were shown in Table 2. The ribosome pathway contained 113 DEGs and took the first place in the list, followed by protein processing in endoplasmic reticulum. Many of the DEGs were mapped to nutritional regulation network. For instance, biosynthesis of amino acids ranked third in the top 20 pathways by containing 30 DEGs, such as fructose 1, 6-bisphosphate aldolase, aspartate aminotransferase and glyceraldehyde-3-phosphate dehydrogenase, which showed infinitely more transcripts at fed status than in starved status. Pathways involved in energy metabilism, like carbon metabolism, glycolysis/gluconeogenesis, glutathione metabolism and purine metabolism, were also in the list (Table 2). Transcripts level of *vitellogenin* (*Vg*) in fed female adult was sharply elevated with log_2_FC>12 compared to that in starved individual. In the ILP/ToR signaling pathway, many elements varied in mRNA abundance between the two nutritious statuses (Table 3). However, for most components, including *ILPs*, their expressions were not significantly affected by nutrition level (Fig 7). Among the three *C*. *pallens ILPs*, *ILP1* and *ILP2* were down-regulated in fed animal, but *ILP3* showed not significant changes. For the two *InRs*, both *InR1* and *InR2* were up-regulated in fed condition (Table 3). Additionally, certain enzymes involved in nutrition degradation and transportation, such as serine protease, was down-regulated in fed individuals (Table 3).

**Table 3:** Expression profiles of genes involved in vitellogenesis, nutrition metabolism and ILP/ToR signaling.

Gene ID	Annotations	Accession No.	FPKM		FC^a^
			Starved	Fed	
CL242Contig1	Vg2 (Vitellogenin 2)	BAA22791.1	0.11	2268.15	15.28
CK1.comp19582_c0_seq1	Vg1 (Vitellogenin 1)	BAA22791.1	0.56	2614.16	12.59
CK1.comp19582_c0_seq3	Vg3 (Vitellogenin 3)	BAJ33507.1	0.21	1807.08	12.97
CL159Contig1	Chymotrypsin	KDR14900.1	30.14	808.01	5.01
T.comp17771_c0_seq2	InR1 (Insulin Receptor 1)	EGI60406.1	1.24	11.38	4.85
CK1.comp16379_c0_seq1	Trypsin	XP_001663894.1	2.52	17.11	3.09
T.comp16502_c0_seq3	PI3K (Phosphoinositide-3-Kinase)	XP_001606345.2	0.14	0.61	2.40
CK1.comp9288_c0_seq1	S6K (Ribosomal Protein S6 Kinase)	XP_003394553.1	2.72	7.14	1.42
T.comp16181_c1_seq1	AKT (serine/threonine-protein kinase)	XP_001601897.1	5.04	10.81	1.39
CK1.comp19518_c0_seq1	InR2 (Insulin Receptor 2)	XP_011062671.1	12.51	25.90	1.32
T.comp9443_c0_seq1	Pten	EZA50068.1	6.85	7.30	0.37
CK1.comp14295_c0_seq1	RHEB (Ras Homolog Enriched in Brain)	XP_003706376.1	2.09	2.62	0.34
T.comp14445_c0_seq1	TSC2 (Tuberous Sclerosis Complex 2)	XP_395739.4	1.33	1.64	0.32
T.comp13784_c0_seq1	ILP3 (Insulin-Like Peptide 3)	CAP09890.1	0.21	0.25	0.25
T.comp7677_c0_seq1	Trehalase	AFK66763.1	5.82	5.23	-0.11
T.comp23554_c0_seq1	ToR (Target of Rapamycin)	ACH47049.1	6.57	4.13	-0.35
CL426Contig1	Chico	XP_006565106.1	12.43	5.40	-0.86
c50851.graph_c0	Serine protease	XP_001653940.1	1058.74	464.02	-0.88
c65318.graph_c0	Sugar transporter	ETN58528.1	108.70	41.43	-1.10
T.comp13721_c0_seq1	FoxO (Fox Head Box Transcription Factor)	NP_001034503.2	0.96	0.43	-1.15
CK1.comp14042_c0_seq2	TSC1 (Tuberous Sclerosis Complex 1)	XP_974036.1	2.80	1.17	-1.23
CL1Contig60	ILP2 (Insulin-Like Peptide 2)	XP_003247548.1	0.34	0.11	-1.31
CK1.comp12348_c0_seq1	ILP1 (Insulin-Like Peptide 1)	XP_001956270.1	0.24	0.06	-2.26
CK1.comp8119_c0_seq1	4E-BP (Translation Initiation Factor 4E-Binding Protein)	XP_003401800.1	62.84	10.88	-2.34
CK1.comp6955_c0_seq1	Lipase	KDR16428.1	2.19	0.00	-Inf

^a^FC, fold change (log_2_ ratio) of gene expression.

### Quantitative real-time PCR validation

**Fig 8:**
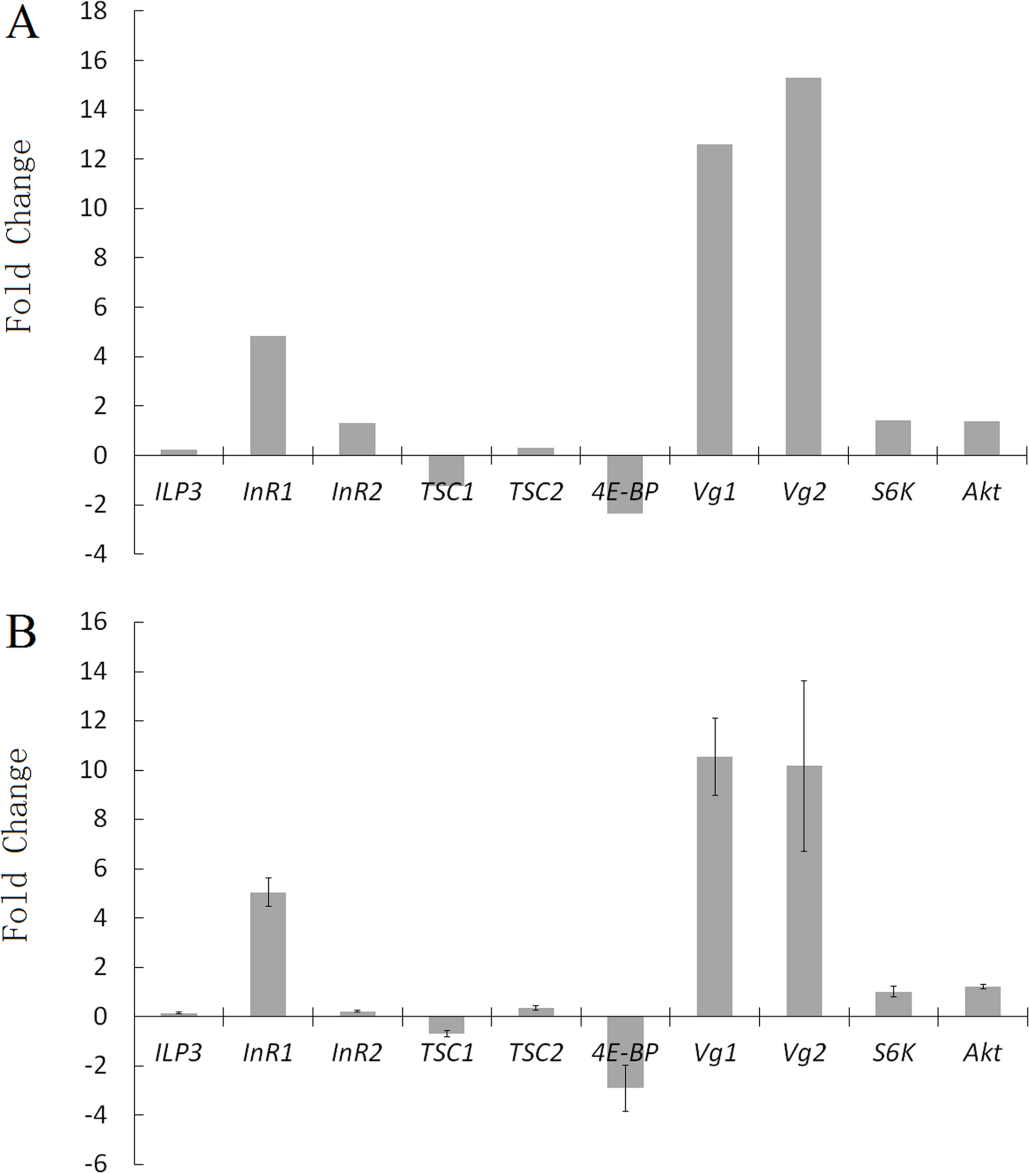
Verification of differentially expressed genes (DEG) in ILP signaling pathway by qRT-PCR. (A) DEG results from transcriptome analysis. The fold change of gene was calculated as log_2_ value of each fed/starved comparison and shown on y-axis. (B) qRT-PCR results of gene expression. Values are expressed as Mean±SE of three independent replicates.

Differently expressed genes (DEGs) were validated by qPCR. Ten component genes involved in Vg synthesis and ILP signaling, including *Vg*, *ILPs*, *InRs*, *Akt*, *S6K*, *4E-BP*, *TSC1* (*tuberous sclerosis complex 1*) and *TSC2* (*tuberous sclerosis complex 2*) were selected and their relative mRNA abundance in starved and fed conditions were monitored. qRT-PCR results were found in agreement with the DEG results, suggesting that transcriptome data are reliable (Fig 8).

## Discussion

The green lacewing, *Chrysopa pallens*, is among the most effective entomophagous predators because of wide range of prey, extensive distribution and excellent predatory performance at both larval and adult stages [28, 29]. In 2013, the first transcriptome analysis in *C*. *pallens* was carried out with an emphasis on chemoreception in antennae [27]. In present study, gene expression between starved and fed conditions was compared by Illumina sequencing to gain insight into the molecular properties of vitellogenesis, energy metabolism and reproduction regulation in *C*. *pallens* female adult. The obtained information will contribute to mass rearing and commercial use of this natural enemy insect.

The illumina sequencing of starved and fed females generated about totally 181 million clean reads, which were further assembled into 71236 unigenes. The N50 of the unigenes is 1615 bp (Table 1), a value much higher than that in previous *C*. *pallens* transcriptome [27], indicating a high quality in sequencing and de novo assembly. BLASTX annotation revealed that *C*. *pallens* shared highest sequence similarity with *T*. *castaneum* (Fig 1). This is not consistent with results implied by phylogenetic trees of ILPs and InRs (Figs 4 and 5). So far, the close evolutionary relationship between Neuroptera and Coleoptera has been confirmed by the prior transcriptome [27] and molecular trees based on 18S rRNA and Vgs [29,30]. As BLASTX results were based on alignment analysis of tens of thousands of sequences, we hypothesize that phylogenetic relationship revealed by species distribution (Fig 1) should be more reliable than that suggested by molecular tree based on a single gene.

The transcriptomes were filtrated for genes in vitellogenesis and ILP signaling pathway. Totally, five unigenes were identified as Vg-encoding sequences in the *C*. *pallens* transcriptomes. In past several decades, the *Vgs* have been studied extensively in several insect orders. Certain species, such as *Leucophaea maderae*, *Plautia stali* and *Aedes aegypti*, possess more than one *Vg* genes [31]. Here, we first showed that the neuropteran *C*. *pallens* also has multiple *Vgs*. Since the 3 *C*. *pallens Vgs* showed similar differences in transcripts level between the two nutritional conditions (Table 3), it is likely that they contribute equally to *C*. *pallens* embryogenesis. The mRNA levels of the three *C*. *pallens Vgs* were much higher in fed females than in starved ones, suggesting that 36 h post eclosion is a good timing for sampling in the illumina sequencing. We did not use older females just because that, in preliminary experiment, *C*. *pallens* could merely survive 2 days after emergence when supplied with water alone.

Sequence annotation revealed that *C*. *pallens* has 3 *ILPs* (Table 3). Up to now, presence of multiple *ILPs* has been demonstrated in *Bombyx mori*, *Nilaparvata lugens*, *Tribolium castaneum*, *Drosophila melanogaster*, *Aedes aegypti*, etc [5,7,10,32,33]. Mammalia insulin and insect ILP belong to secretory protein and are characterized by a signal peptide at the N-end of pre-propeptide [5]. However, SignalP 4.1 predicted that *C*. *pallens* ILP3 contains no this structure. An ILP without signal peptide may have different function and mechanism. Characterization of *C*. *pallens* ILPs is the first step toward synthesis of bioactive ILP molecular and application in mass rearing. It has been reported that increase of ILP signals triggered *Vg* expression in a few species, such as *T*. *castaneum* [7]. In a previous experiment, injection of bovine insulin into previtellogenic *C*. *pallens* significantly promoted vitellogenin synthesis and egg production [34]. We infer that *C*. *pallens* ILP will exhibit promising results in stimulating lacewing vitellogenesis and fecundity, because ILPs are much more efficient than mammalian insulin when applied in insects [35]. Due to the crucial roles in regulation of various physiological processes, ILPs will show us attractive potential in artificial regulation of insect reproduction, diapause, longevity, etc.

The *C*. *pallens* transcriptomes displayed an insulin signaling pathway characterized by up-regulation of *InR1 InR2*, down-regulation of *CpILP1*, *CpILP2*, and unchanged expression of most other elements at fed station (Fig 7). The reverse expression patterns of *C*. *pallens ILPs* at the same nutritional condition suggested their different functions. It is very common that certain insect species have multiple ILPs [5]. *N*. *lugen* has four ILPs, but only NlILP3 is crucial in determination of wing morphs [33]. In *Anopheles gambiae*, different *ILPs* have diverse transcripts level at different developmental stages [36]. *CpILP1* and *CpILP2* were down-regulated is just because that these two ILPs are not necessary in sense and transduction of nutrition signals at the beginning of adult stage. Transcriptome analysis revealed that *C*. *pallens* has two *InRs*. Just like InRs in *N*. *lugens*, the two *C*. *pallens* InRs have very low amino acid sequence identity (22.45%). Unlike the three *C*. *pallens ILPs*, both *C*. *pallens InR1* and *InR2* were up-regulated at fed condition (Table 3). In previous reports, *InR* always increase expression when sufficient nourishment is available [18,37]. The sharp increase of *InR1* expression at fed condition led us to conclude that *InR1* is the key player in nutritional and reproductive signaling of *C*. *pallens* female adult. Recently, the *N*. *lugens InRs* deepened our understanding of the development and evolution of phenotypic plasticity with a binary control over alternative wing morphs [33]. The roles and mechanism of the *C*. *pallens InRs* needs to be further explored. Aside from *ILP*, other components, such as *FoxO*, also showed no significant changes in transcripts abundance between starved and fed *C*. *pallens*. FoxO is a subgroup of the forkhead-box family of transcription factors. In feeding condition, FoxO is phosphorylated and remains inactive in the cytoplasm. However, in a state of nutrient restriction, unphosphorylated FoxO translocates to nucleus to exert its transcriptional action, resulting an arrest of reproductive process [19,21]. In *B*. *germinica*, *FoxO* is also not nutritionally regulated because it showed no significant differences in mRNA level between fed and starved individuals [19]. In addition to *FoxO*, *4E-BP* also showed no significant difference in mRNA level between the two nutritional conditions. 4E-BP, the translation initiation factor 4E-binding protein, encodes a family of translation repressor proteins. The unphosphorylated protein directly interacts with eukaryotic translation initiation factor 4E (eIF4E) and represses translation. On the contrary, phosphorylation of 4E-BP leads to its dissociation from eIF4E and activation of mRNA translation [38]. The unchanged expression level of these elements in starved and fed *C*. *pallens* suggested that they transduct ILP signals by phosphorylation, independent of nutritious status. Among the top 20 KEGG pathways sorted by DEG number (Table 2), the ribosome, protein processing and biosynthesis of amino acids in endoplasmic reticulum ranked the first, second and third, respectively, suggesting a sharp difference in peptide synthesis and protein assembly between the two nutritional statuses. In addition, glycolysis/gluconeogenesis ranked sixth in the list by containing 22 DEGs, indicating an active glycometabolism in the fed green lacewing.

The expression profiles of enzymes involved in energy metabolism and transportation were compared between different nutritional conditions of *C*. *pallens*. Similar to results revealed by microarray analysis in *T*. *castaneum* [18], the expression of some digestive enzymes, like serine protease, was decreased under feeding condition (Table 3). We inferred that this is because serine protease plays multiple physiological roles in insect, including digestion, emostasis, apoptosis, signal transduction, reproduction, and the immune response [39]. Maybe, other functions beyond digestion are not required in fed condition during the pre-oviposition period. So, decrease of total serine protease mRNA does not necessarily mean a reduction of enzyme participating in protein digestion.

## Materials and methods

### Insect rearing

The *C*. *pallens* culture was fed with pea aphid (*Acyrthosiphon pisum*) reared on broad bean (*Vicia faba* L.) at 25°C, 70% relative humidity under a 16:8 (L:D) photoperiod. The initial lacewing colonies were kindly provided by Dr. Zhang Fan, Beijing Academy of Agriculture and Forestry Sciences and maintained for more than 30 generations at the controlled conditions. Two groups of female lacewing were prepared. One group is fed with water and starved for 36 h continuously after emergence; the other group is fed on prey continuously for 36 h post emergence. Females and males (1:2) were put together to guarantee sufficient mating. Total RNAs isolated from these two groups were used for illumina sequencing as described below.

### RNA isolation, cDNA library preparation and Illumina sequencing

36 h post emergence, total RNAs was extracted from whole body of *C*. *pallens* females using TRIzol Reagent (Invitrogen, Carlsbad, CA, USA) according to manufacturer’s instructions. Four individuals were used for RNA preparation in each feeding condition. RNA samples were purified from 20 mg of pooled total RNA using Oligo (dT) magnetic beads and interrupted into short fragments in fragmentation buffer. First-strand cDNA was synthesized using Oligo (dT) and second-strand cDNA was then generated. After end-repair and adaptor ligation, PCR was performed and products were purified with QIAquick PCR extraction kit (Qiagen, Venlo, Netherlands) to establish 8 cDNA libraries. Sequencing of the library was carried out on an Illumina HiSeq™ 2500 platform.

### Sequence assembly and function annotation

Raw reads were generated using Solexa GA pipeline 1.6. After removal of low quality reads, filtered reads were assembled with Trinity program. All transcriptome raw data was submitted to NCBI SRA database. Unigenes larger than 150 bp were aligned by BLASTX to protein databases such as Non-redundant (Nr), Swiss-Prot, KEGG and COG (e-value<10^−5^) and BLASTN to the NCBI nucleotide databases (e-value<10^−5^) to retrieve proteins with the maximum sequence identity with the given unigenes along with functional annotations. Containing of signal peptide was predicted by SignalP 4.1 (http://www.cbs.dtu.dk/services/SignalP/).

### Analysis of differential gene expression

Differentially expressed genes (DEGs) between two nutritional stations were identified by a rigorous algorithm according to the methods described previously [40]. Relative transcript levels were output as RPKM (Reads Per Kilobase of exon model per Million mapped reads) value. Threshold *P*-value in multiple test and analysis was determined by false discovery rate (FDR). An FDR<0.001 and an absolute value of the log_2_ ratio ≥1 were used to determine the significance of gene expression difference [41].

### Sequence alignment and phylogenetic analysis

The amino acid alignment of the ILP, InR, ToR candidates were carried out using LCUSTALX 2.0 and arranged by Jalview 2.4.0 b2. The 3 Vgs, 3 ILPs and 2 InRs sequences from the adult transcriptome of *C*. *pallens*, along with their counterparts from other insect species were used to construct phylogenetic trees. The Vg data set contained 75 Vgs from 62 other insect species (S1 Table). The ILP data set contained 76 ILPs from 19 other insect species (S2 Table). The InR data set contained 47 InRs from 28 other insect species (S3 Table).

### Real-time PCR analysis

To confirm the RPKM results, the transcription levels of 10 genes in ILP and ToR signaling pathway was tested at two nutritious statuses by qRT-PCR. Total RNAs were extracted from single adult female using Tranzol reagents (Transgene, Beijing, China) and treated with RNase-free DNase (Takara, Kyoto, Japan). cDNA was synthesized using TransScript First-Strand cDNA Synthesis SuperMix (Transgene, Beijing, China) with anchored Oligo(dT)18 primer and M-MLV reverse transcriptase. qRT-PCR was carried out in a 20 μl reaction system containing 200 nM each of forward and reverse gene specific primers, 10 μl of 2×SYBR Green Real-time PCR Master Mix (Toyobo, Shanghai, China), cDNA produced from 2 μg total RNA. The housekeeping gene *C*. *pallens actin* was used as internal control for normalization [29]. Sequence specific primers were designed with Primer Premier 5.0 and shown in S4 Table. The quantitative validation was analyzed by the 2^-ΔΔCt^ method [42]. Means and standard errors for each time point were obtained from the average of three independent sample sets.

## Supporting information

S1 Table Vitellogenins (Vgs) used in phylogenetic tree construction, including protein name and GenBank accession number. (DOCX)

S2 Table Insulins and Insulin-Like Peptides (ILPs) used in phylogenetic tree construction, including protein name and GenBank accession number. (DOCX)

S3 Table Insulin Receptors (InRs) used in phylogenetic tree construction, including protein name and GenBank accession number. (DOCX)

S4 Table Sequences of qPCR primers. (DOCX)
