# Supplementary material for: Nutritional and reproductive signaling revealed by comparative gene expression analysis in Chrysopa pallens (Rambur) at different nutritional statuses
Source: PLoS One. 2017 Jul 6;12(7):e0180373. doi: 10.1371/journal.pone.0180373 (PMC5500325; doi:10.1371/journal.pone.0180373)
Supplement: S1 Table — (DOCX) [file pone.0180373.s001.docx]

S1 Table. Vitellogenins (Vgs) used in phylogenetic tree construction, including protein name and GenBank accession number.

| Species | Protein name | GenBank accession number | Species | Protein name | GenBank accession number |
| --- | --- | --- | --- | --- | --- |
| *Actias selene* | Vg1 | ABP63663.1 | *Habropoda laboriosa* | Vg | KOC66503.1 |
| *Actias selene* | Vg2 | ADB94560.1 | *Harpegnathos saltator* | Vg1 | EFN75537.1 |
| *Aedes aegypti* | Vg1 | AAA18221.1 | *Harpegnathos saltator* | Vg2 | EFN86099.1 |
| *Aedes aegypti* | Vg2 | AAA99486.1 | *Helicoverpa armigera* | Vg1 | AFV40972.1 |
| *Anopheles minimus* | Vg | AHN13887.1 | *Helicoverpa armigera* | Vg2 | AGL08685.1 |
| *Anopheles subpictus* | Vg | AHM10344.1 | *Homalodisca vitripennis* | Vg | AAZ06771.1 |
| *Antheraea pernyi* | Vg | BAB16412.1 | *Laodelphax striatella* | Vg1 | AGJ26477.1 |
| *Antheraea yamamai* | Vg | BAB32640.1 | *Laodelphax striatella* | Vg2 | AGJ26478.1 |
| *Anthonomus grandis* | Vg | AAA27740.1 | *Lethocerus deyrollei* | Vg | BAG12118.1 |
| *Apis cerana* | Vg | NP_001315413 | *Lygus hesperus* | Vg | JAG28748.1 |
| *Apis mellifera* | Vg | CAD56944.1 | *Lymantria dispar* | Vg | AAB03336.1 |
| *Apolygus lucorum* | Vg1 | AFW97644.1 | *Nasonia vitripennis* | Vg | XP_001607388.1 |
| *Apolygus lucorum* | Vg2 | AGT39945.1 | *Nesidiocoris tenuis* | Vg | AGV05363.1 |
| *Athalia rosae* | Vg | BAA22791.1 | *Nilaparvata lugens* | Vg1 | BAF75351.1 |
| *Bactrocera latifrons* | Vg1 | JAI20767.1 | *Nilaparvata lugens* | Vg2 | AEL22916.1 |
| *Bactrocera latifrons* | Vg2 | JAI30045.1 | *Nilaparvata lugens* | Vg3 | BAP87098.1 |
| *Bemisia tabaci* | Vg1 | ADU04392.1 | *Octodonta nipae* | Vg | AKR04341.1 |
| *Bemisia tabaci* | Vg2 | ADU04393.1 | *Operophtera brumata* | Vg | KOB76724.1 |
| *Blattella germanica* | Vg | CAA06379.2 | *Osmia cornifrons* | Vg | AIU68826.1 |
| *Bombus hypocrita* | Vg | ACU00433.1 | *Papilio machaon* | Vg | KPJ19580.1 |
| *Bombus ignitus* | Vg1 | ACM46019.1 | *Papilio xuthus* | Vg | KPJ04900.1 |
| *Bombus ignitus* | Vg2 | ACQ91623.1 | *Periplaneta americana* | Vg | BAA86656.1 |
| *Bombyx mandarina* | Vg1 | BAB32642.2 | *Pimpla nipponica* | Vg | AAC32024.1 |
| *Bombyx mandarina* | Vg2 | BAE47146.1 | *Pteromalus puparum* | Vg | ABO70318.1 |
| *Bombyx mori* | Vg | BAA02444.1 | *Rhynchophorus ferrugineus* | Vg | ALN38803.1 |
| *Cadra cautella* | Vg | ALN38805.1 | *Rhyparobia maderae* | Vg | BAB19327.1 |
| *Camponotus floridanus* | Vg1 | EFN64902.1 | *Riptortus clavatus* | Vg | AAB72001.1 |
| *Camponotus floridanus* | Vg2 | EFN69845.1 | *Samia cynthia pryeri* | Vg | BAD91196.1 |
| *Chilo suppressalis* | Vg | AMD78107.1 | *Samia ricini* | Vg | BAB32641.1 |
| *Chrysopa pallens* | Vg | AGJ71349.1 | *Saturnia japonica* | Vg | BAD91195.1 |
| *Cimex lectularius* | Vg | BAU36889.1 | *Solenopsis invicta* | Vg | AAP47155.1 |
| *Cnaphalocrocis medinalis* | Vg | AEM75020.1 | *Spodoptera exigua* | Vg | ALR35190.1 |
| *Corcyra cephalonica* | Vg | AHZ89334.1 | *Spodoptera litura* | Vg | ABU68426.1 |
| *Danaus plexippus* | Vg | EHJ67298.1 | *Tenebrio molitor* | Vg | AAU20328.2 |
| *Encarsia formosa* | Vg | AAT48601.1 | *Tribolium castaneum* | Vg1 | XP_971398.1 |
| *Fopius arisanus* | Vg | JAG72420.1 | *Tribolium castaneum* | Vg2 | XP_970210.1 |
| *Formica exsecta* | Vg | AII96912.1 | *Trigonotylus caelestialium* | Vg | BAJ33507.1 |
| *Geocoris pallidipennis* | Vg | ALN70475.1 | *Vespula vulgaris* | Vg | AER70365.1 |
| *Graptopsaltria nigrofuscata* | Vg | BAA85987.1 |  |  |  |
