# Supplementary material for: Nutritional and reproductive signaling revealed by comparative gene expression analysis in Chrysopa pallens (Rambur) at different nutritional statuses
Source: PLoS One. 2017 Jul 6;12(7):e0180373. doi: 10.1371/journal.pone.0180373 (PMC5500325; doi:10.1371/journal.pone.0180373)
Supplement: S2 Table — (DOCX) [file pone.0180373.s002.docx]

| Protein name | GenBank accession number | Protein name | GenBank accession number |
| --- | --- | --- | --- |
| AaILP1 | ABI64116.1 | BmB12 | NP_001121794.1 |
| AaILP2 | ABI64118.2 | BmC1 | NP_001119736.1 |
| AaILP3 | ABI64117.2 | BmC2 | NP_001119735.1 |
| AaILP4 | ABI64119.1 | BmD1 | NP_001121635.1 |
| AaILP5 | ABI64124.1 | BmE1 | NP_001119733.1 |
| AaILP6A | ABI64121.1 | BmF1 | NP_001119734.1 |
| AaILP6B | ABI64122.1 | BmG1 | NP_001121634.1 |
| AaILP7 | ABI64123.1 | BtILP | ACD35246.1 |
| AaILP8 | ABI64120.1 | CeILP | AAC33275.1 |
| AgILP1 | AAQ89692.1 | CgILP | XP_011455161.1 |
| AgILP2 | AAQ89693.1 | CpILP2 | AC |
| AgILP3 | AAQ89694.1 | CqIL6P | EDS39071.1 |
| AgILP4 | AAQ89695.1 | CqILP1 | EDS26450.1 |
| AgILP5 | AAQ89697.1 | CqILP5 | XP_001843238.1 |
| AgILP6 | AAQ89698.1 | DmILP1 | NP_648359.1 |
| AgILP7 | AAQ89700.1 | DmILP2 | NP_524012.1 |
| BcILP | AAA62720.1 | DmILP3 | AAF50203.2 |
| BmA1 | Q17192.1 | DmILP4 | NP_648361.1 |
| BmA2 | NP_001103771.1 | DmILP5 | NP_996037.1 |
| BmA3 | NP_001166890.1 | DmILP6D | NP_001259185.1 |
| BmA4 | NP_001121607.1 | DmILP7 | NP_570070.1 |
| BmA5 | NP_001121608.1 | DnILP | KZC07709.1 |
| BmA6 | BAA00667.1 | EmILP | OAD57357.1 |
| BmA7 | NP_001121629.1 | HlILP | KOC68498.1 |
| BmA8 | NP_001121630.1 | HsILP | AAA59172.1 |
| BmA9 | BAA00672.1 | LmILP | CAA34889.1 |
| BmA10 | NP_001121787.1 | MqILP | KOX71094.1 |
| BmB1 | NP_001121791.1 | NlILP1 | AIY24645.1 |
| BmB2 | NP_001121793.1 | NlILP2 | AIY24646.1 |
| BmB3 | BAA00674.1 | NlILP3 | AIY24647.1 |
| BmB4 | NP_001121792.1 | NlILP4 | AIY24648.1 |
| BmB5 | NP_001166891.1 | NlILP5 | BAO00957.1 |
| BmB6 | NP_001121795.1 | NlILP6 | BAO00958.1 |
| BmB7 | NP_001121789.1 | PcILP | XP_014665770. |
| BmB8 | NP_001121790.1 | TcILP | EEZ99258.2 |
| BmB9 | BAA00682.1 | TcILP | EFA02796.2 |
| BmB10 | NP_001121788.1 | TcILP | EFA02918.1 |
| BmB11 | NP_001121606.1 | ZnILP | KDR22482.1 |

S2 Table. Insulins and Insulin-Like Peptides (ILPs) used in phylogenetic tree construction, including protein name and GenBank accession number.
