# Supplementary material for: Nutritional and reproductive signaling revealed by comparative gene expression analysis in Chrysopa pallens (Rambur) at different nutritional statuses
Source: PLoS One. 2017 Jul 6;12(7):e0180373. doi: 10.1371/journal.pone.0180373 (PMC5500325; doi:10.1371/journal.pone.0180373)
Supplement: S3 Table — (DOCX) [file pone.0180373.s003.docx]

S3 Table. Insulin Receptors (InRs) used in phylogenetic tree construction, including protein name and GenBank accession number.

| Species | Protein name | GenBank accession number | Species | Protein name | GenBank accession number |
| --- | --- | --- | --- | --- | --- |
| *Acyrthosiphon pisum* | InR | XP_001942660.2 | *Drosophila suzukii* | InR | XP_016926862.1 |
| *Apis cerana* | InR | XP_016916506.1 | *Dufourea novaeangliae* | InR | XP_015436217.1 |
| *Apis dorsata* |  | XP_006612442.1 | *Eufriesea mexicana* | InR | OAD59778.1 |
| *Apis dorsata* |  | XP_006618839.1 | *Fopius arisanus* | InR | JAG75546.1 |
| *Apis florea* | InR | XP_003690408.2 | *Habropoda laboriosa* | InR | KOC65570.1 |
| *Apis mellifera* | InR | XP_394771.5 | *Harpegnathos saltator* | InR | EFN85558.1 |
| *Atta colombica* | InR | KYM78696.1 | *Lasius niger* | InR | KMQ89997.1 |
| *Bactrocera dorsalis* | InR | XP_011202029.1 | *Megachile rotundata* | InR | XP_003700128.1 |
| *Bactrocera latifrons* | InR | JAI45869.1 | *Melipona quadrifasciata* | InR | KOX67662.1 |
| *Bactrocera oleae* | InR | XP_014086543.1 | *Papilio machaon* | InR1 | KPJ07067.1 |
| *Bombus impatiens* | InR1 | XP_003484422.1 | *Papilio machaon* | InR2 | XP_014355372.1 |
| *Bombus impatiens* | InR2 | XP_003490625.1 | *Papilio polytes* | InR | XP_013145759.1 |
| *Bombus impatiens* | InR3 | XP_012245698.1 | *Papilio xuthus* | InR | XP_013173086.1 |
| *Bombus terrestris* | InR1 | XP_003393794.1 | *Pogonomyrmex barbatus* | InR1 | XP_011648401.1 |
| *Bombus terrestris* | InR2 | XP_003397946.1 | *Pogonomyrmex barbatus* | InR2 | XP_011648405.1 |
| *Camponotus floridanus* | InR1 | EFN73169.1 | *Polistes canadensis* | InR | XP_014598475.1 |
| *Camponotus floridanus* | InR2 | XP_011263722.1 | *Polistes dominula* | InR | XP_015183031.1 |
| *Cephus cinctus* | InR | XP_015593940.1 | *Solenopsis invicta* | InR | NP_001291521.1 |
| *Ceratosolen solmsi marchali* | InR | XP_011494410.1 | *Stomoxys calcitrans* | InR | XP_013109366.1 |
| *Cyphomyrmex costatus* | InR | KYN05428.1 | *Trachymyrmex cornetzi* | InR | KYN16995.1 |
| *Diaphorina citri* | InR1 | XP_008471421.1 | *Trachymyrmex septentrionalis* | InR | KYN36302.1 |
| *Diaphorina citri* | InR2 | XP_008479213.1 | *Wasmannia auropunctata* | InR | XP_011690939.1 |
| *Dinoponera quadriceps* | InR | XP_014476870.1 | *Zootermopsis nevadensis* | InR | KDR10688.1 |
| *Diuraphis noxia* | InR | XP_015363980.1 |  |  |  |
