# Supplementary material for: Nutritional and reproductive signaling revealed by comparative gene expression analysis in Chrysopa pallens (Rambur) at different nutritional statuses
Source: PLoS One. 2017 Jul 6;12(7):e0180373. doi: 10.1371/journal.pone.0180373 (PMC5500325; doi:10.1371/journal.pone.0180373)
Supplement: S4 Table — (DOCX) [file pone.0180373.s004.docx]

| Unigene | *Annotation* | Forward | Reverse | Product size (bp) |
| --- | --- | --- | --- | --- |
| CK1.comp14042_c0_seq1 | *TSC1* | AAAGATTGGCATGTAGCA | ATCCTCGTTATGTGGTTC | 243 |
| T.comp14445_c0_seq1 | *TSC2* | CCAGTTAGGCTTGATGCT | AATGCTGAACAGGGTGAA | 137 |
| CL242Contig1 | *Vg2* | TTGATAGCGATAGCAGTG | GGGCATGGGTTGGTAGTC | 161 |
| T.comp15327_c1_seq2 | *S6K* | CAATCTACCATTTCAGGGAC | CATCATCGGCACGACTAA | 263 |
| T.comp13784_c0_seq1 | *ILP3* | TACAATGGGCATGTGATG | GTGTACGAAATGGAAACG | 135 |
| CL1Contig60 | *ILP2* | TCGTCGATATTGTGGTGA | CACGTCCATGTAGCGTTG | 110 |
| CK1.comp14726_c0_seq1 | *4E-BP* | CGTTGGCTAATATCATTG | AACAGCTCCACATACTTC | 123 |
| CK1.comp22619_c0_seq5 | *InR1* | TTATGTTTCAATGGGCTTCA | TGCGGTTCCAATCCTCTAC | 157 |
| CK1.comp19518_c0_seq1 | *InR2* | GTCAACCGACCTTAGTCA | ATATGCCATACCATCAGC | 176 |
| T.comp16181_c1_seq1 | *Akt* | AATAATGCCAACCGATAAAC | ATCCATTCTTCACGCTCA | 117 |
| / | *Actin* | TCCAGAAGAACACCCAATCC | ATACACCATCACCAGAGTCAAGT | 196 |

S4 Table. Sequences of qPCR primers.
